# Supplementary material for: TET2 mutations are associated with hypermethylation at key regulatory enhancers in normal and malignant hematopoiesis
Source: Nat Commun. 2021 Oct 18;12:6061. doi: 10.1038/s41467-021-26093-2 (PMC8523747; doi:10.1038/s41467-021-26093-2)
Supplement: Supplementary file 1 — Supplementary Information [file 41467_2021_26093_MOESM1_ESM.pdf]

Supplementary Information to:

TET2 mutations are associated with hypermethylation at key regulatory enhancers in normal and malignant hematopoiesis

Supplementary methods 1: Sensitivity analyses on the impact of smoking

Since smoking has been associated both with risk of CHIP[1] and DNA methylation changes[2], we carried out sensitivity analyses to rule out confounding from smoking status/tobacco exposure. DNA methylation is mainly affected by current smoking status, and most methylation changes revert with smoking cessation. Theoretically, CHIP risk should be expected to increase with cumulative tobacco exposure, although some studies have found current smoking status to be more predictive of CHIP. We therefore carried out two sensitivity analyses: One with adjustment for cumulative tobacco exposure with individuals divided into four groups (never-smokers as reference group, and the rest divided into tertiles according to number of pack-years), and one with adjustment for current smoking (yes/no). Smoking history was obtained using a questionnaire at the time of blood collection.

Supplementary methods 2: Hypermethylated regions at transcription start sites (TSS)

We searched for potential hypermethylated TSS regions in *TET2* mutated CHIP, CCUS, and AML with the *comb-p* algorithm[3] using recommended settings[4]. Since *comb-p* combines P values to find differentially methylated regions regardless of effect direction (i.e., a region might be a combination of hypo- and hypermethylated sites), we set the P value of all negatively associated sites to 1. We considered all identified hypermethylated regions with an uncorrected P value < 0.05 and identified 1,396 regions overlapping between the CHIP dataset and the two CCUS datasets. Of these, only 5 were annotated as active TSS based on chromatin states in monocytes, and of these only one region consisting of two CpG sites was located in an actual transcription start site (Supplementary Table 6). Since *comb*-p found no significantly hypermethylated regions in AML, we could not search for overlaps between AML and CHIP or CCUS.

Supplementary Table 1: Age and sex distribution of included individuals with CHIP.

		CHIP	No CHIP
		(N = 116)	(N = 189)
Sex	Men	36 (31%)	60 (32%)
	Women	80 (69%)	129 (68%)
Age (years)	73-75	22 (19%)	32 (17%)
	75-80	57 (49%)	104 (55%)
	80-85	21 (18%)	41 (22%)
	85-90	16 (14%)	12 (6%)

Supplementary Table 2: Baseline characteristics of CCUS patients and healthy controls (granulocyte data). Numbers displayed are counts or median (range).

	CCUS w. *TET2* mut	Healthy controls
Men	1	4
Women	4	4
Age (years)	72 (63–82)	59 (55–65)

Supplementary Table 3: Baseline characteristics of CCUS patients (MNC data). Numbers displayed are counts or median (range).

	CCUS with *TET2* mut	CCUS without *TET2* mut
Men	8	2
Women	2	8
Age (years)	75 (60–82)	68 (54–86)

Supplementary Table 4: Hypermethylated TSS probes as identified using *comb-p* and searching for overlaps between CHIP and CCUS data.

probe	chr	position	Annotated genes
cg23282441	chr10	73533927	C10orf54 (TSS); CDH23 (Body)
cg24499627	chr10	73533891	C10orf54 (TSS); CDH23 (Body)

Supplementary Table 5: Prediction of *TET2* mutations by leave-one-out cross validation in CHIP.

	TET2 mutated (observed)	*TET2* wildtype (observed)
TET2 mutated (predicted)	11	0
TET2 wildtype (predicted)	33	261

Supplementary Table 6: Prediction of *TET2* mutations in CCUS patients and healthy controls

	TET2 mutated (observed)	*TET2* wildtype (observed)
TET2 mutated (predicted)	9	0
TET2 wildtype (predicted)	6	18

Supplementary Table 7: Targeted genes and regions on sequencing panel (coordinates are hg19)

Gene	Chromosome	Start	Stop
ASXL1	20	31025234	31021081
ASXL2	2	25964888	26101101
DNMT3A	2	25457124	25536945
IDH1	2	209113424	209112994
IDH2	15	90631736	90632009
PPM1D	17	58740350	58740919
RAD21	8	117859733	117866713
SF3B1	2	198266563	198267800
SRSF2	17	74732237	74733248
TET2	4	106155075	106197718
TP53	17	7572833	7579961
JAK2	9	5073674	5073808
GNB1	1	Full coding sequence	
GNAS	20	57415162	57415896
	20	57478578	57480535
	20	57484212	57485886
ETV6	12	11803056	12043986
CREBBP	16	3786120	3786810
	16	3781300	3781802
NRAS	1	115256421	115256599
	1	115258671	115258781
KRAS	12	25380168	25380346
	12	25398208	25398318
CBL	11	Full coding sequence	
BRCC3	X	154299797	154348431
BCOR	X	39911356	39937188

Supplementary Figure 1: Waterfall plot showing CHIP mutations in twin cohort.

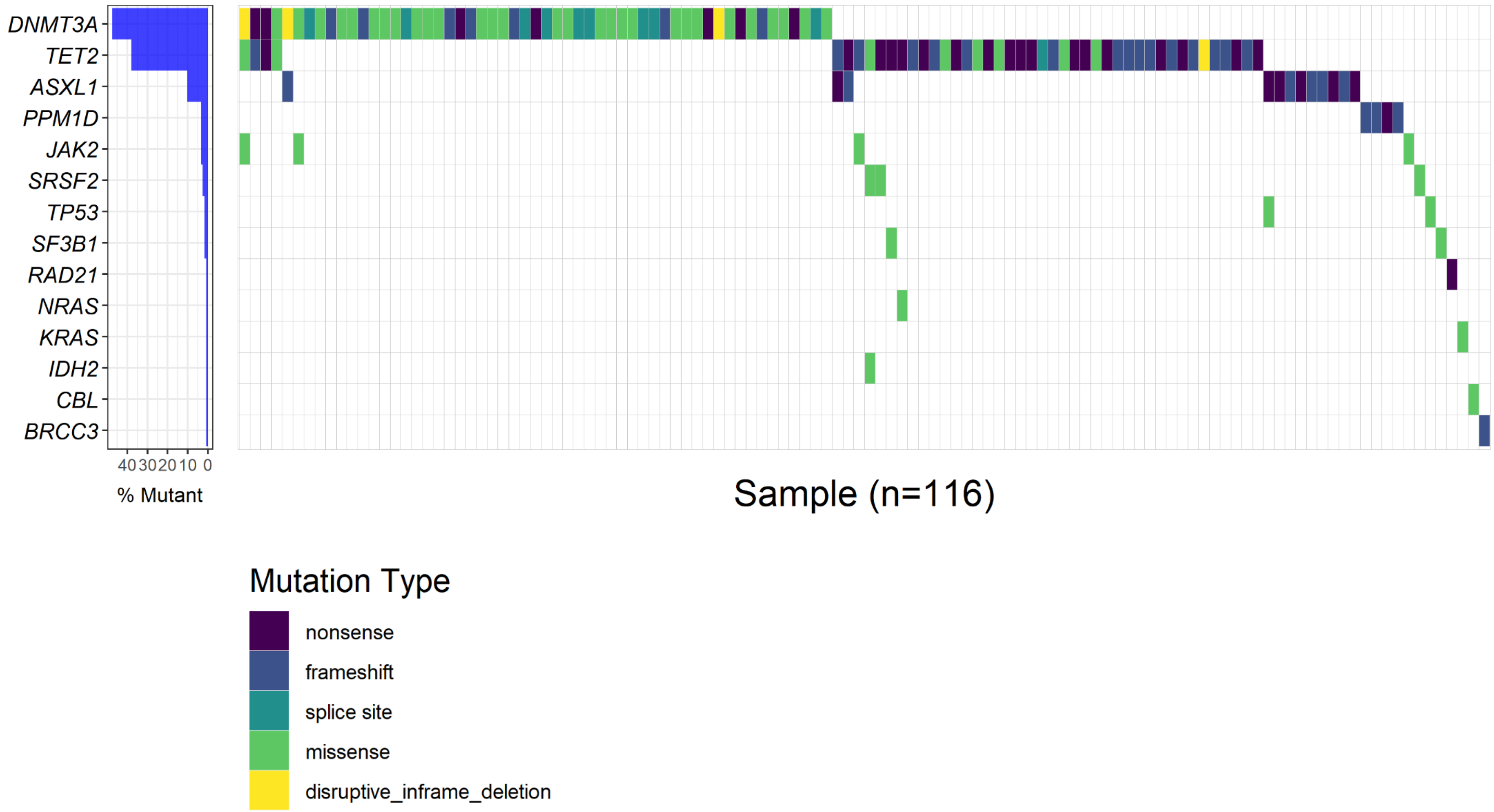

Supplementary Figure 2: Distribution of *TET2* variant allele frequencies. Stacked bars indicate the presence of more than one *TET2* mutation in the given individual.

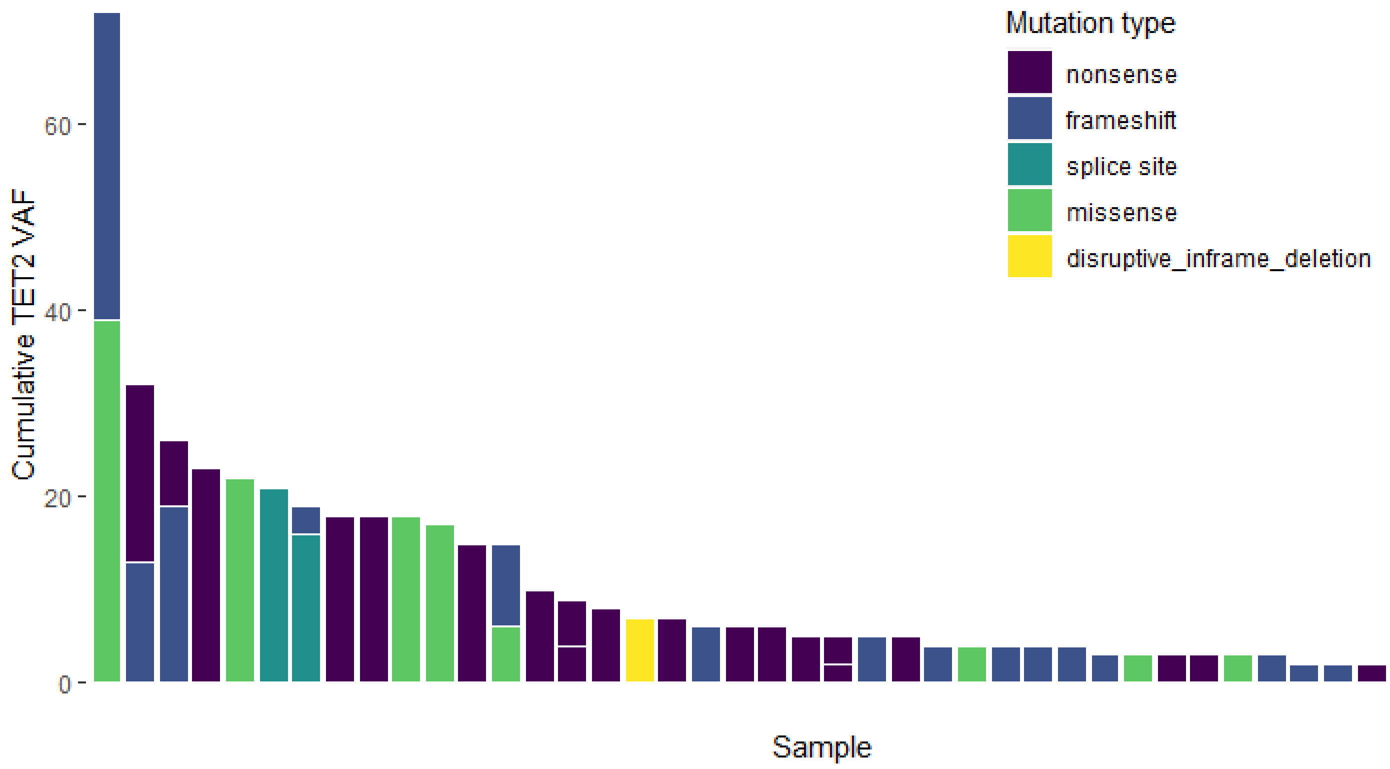

Supplementary Figure 3: Distribution of *DNMT3A* variant allele frequencies in CHIP cohort. Stacked bars indicate the presence of more than one *DNMT3A* mutation in the given individual.

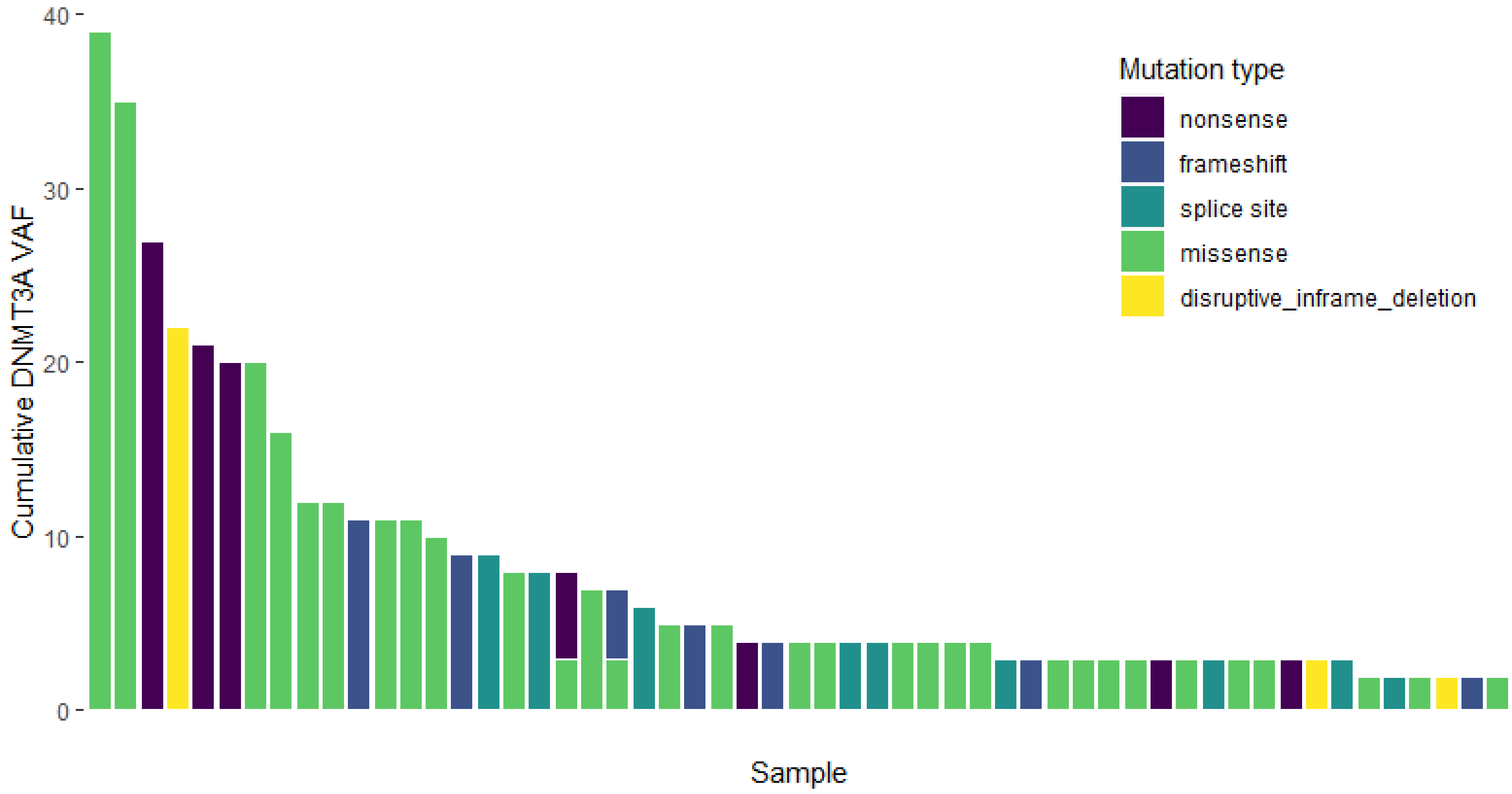

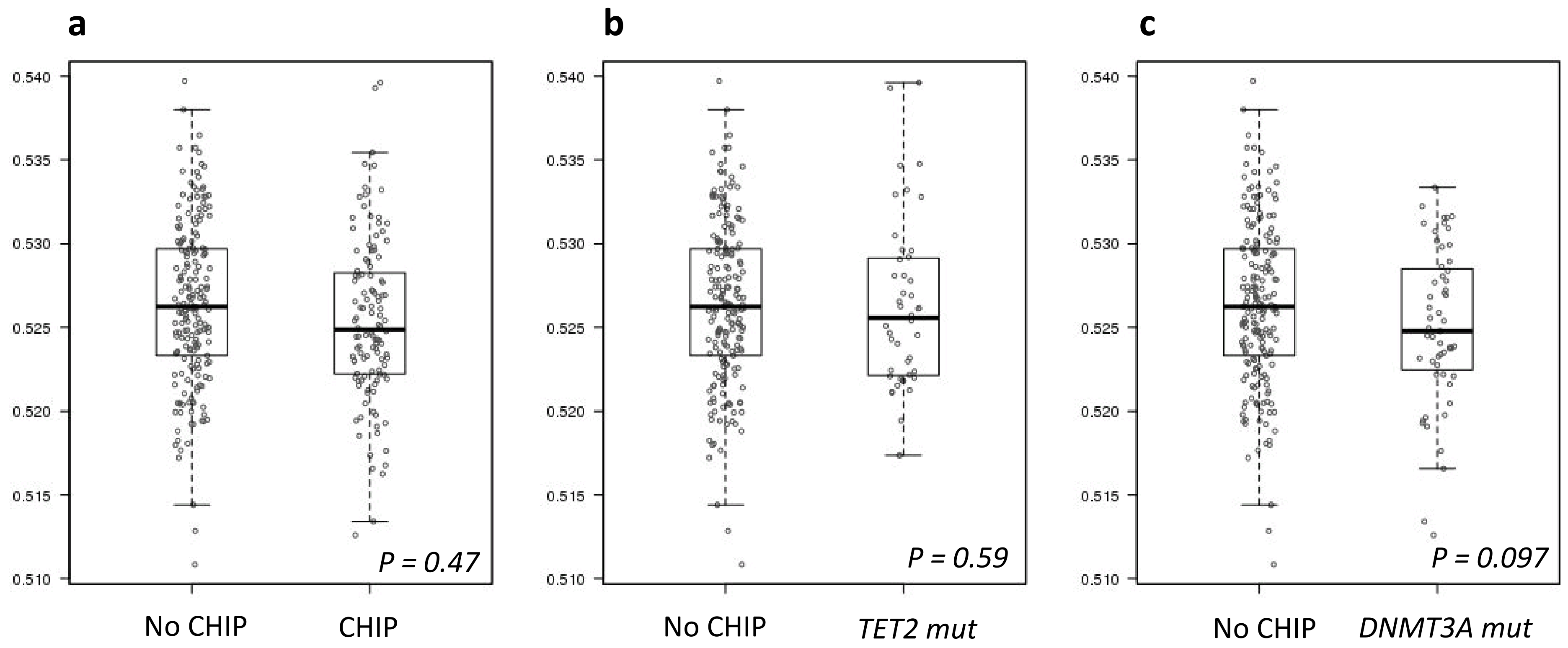

Supplementary Figure 4: Global average methylation levels (beta values) at 427,112 CpG sites. **a** Individuals with (N = 116) vs. without CHIP (N = 189). **b** Individuals with *TET2* mutations (N = 44) vs. individuals without CHIP (N = 189). **c** Individuals with *DNMT3A* mutations (N = 55) vs. individuals without CHIP (N = 189). All P values obtained from a linear mixed effects regression with average methylation level as outcome and mutation status as explanatory variable, with twin pair included as random intercept. Thick lines in box plots represent medians. Boxes represent the interquartile range (IQR). Whiskers represent the most extreme values within 1.5 × IQR.

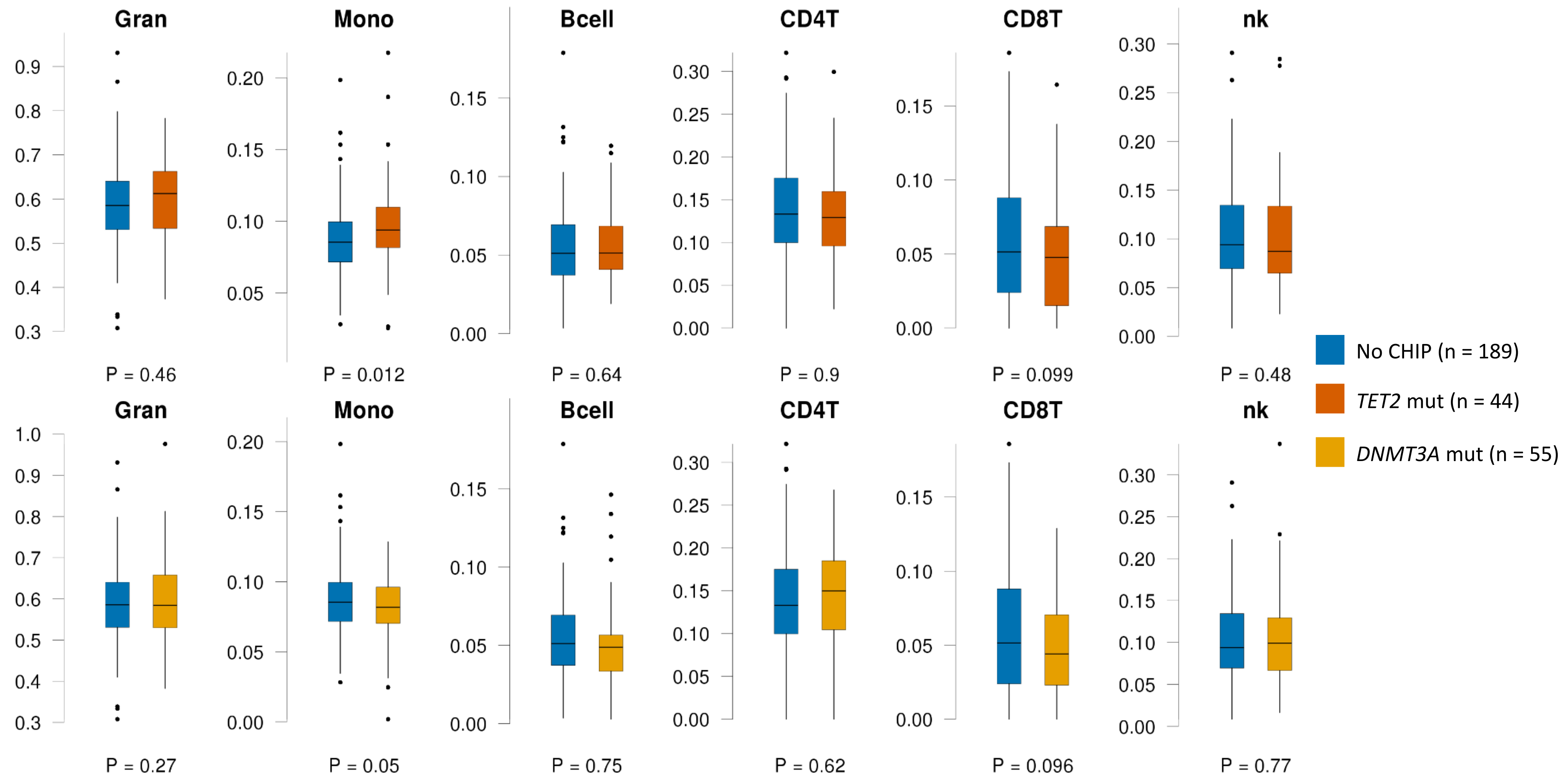

Supplementary Figure 5: Inferred immune cell proportions in individuals with and without *TET2*, or *DNMT3A* mutations. Numbers inferred using the Houseman algorithm[5]. Beta estimates and P values are results from a multivariate linear mixed effects regression with cell count as the outcome and CHIP (or *TET2*/*DNMT3A* mutation) as explanatory variable with adjustment for age, sex, and LSADT cohort and with twin pair as random intercept. Thick lines in box plots represent medians. Boxes represent the interquartile range (IQR). Whiskers represent the most extreme values within 1.5 × IQR, and values outside this range are plotted individually.

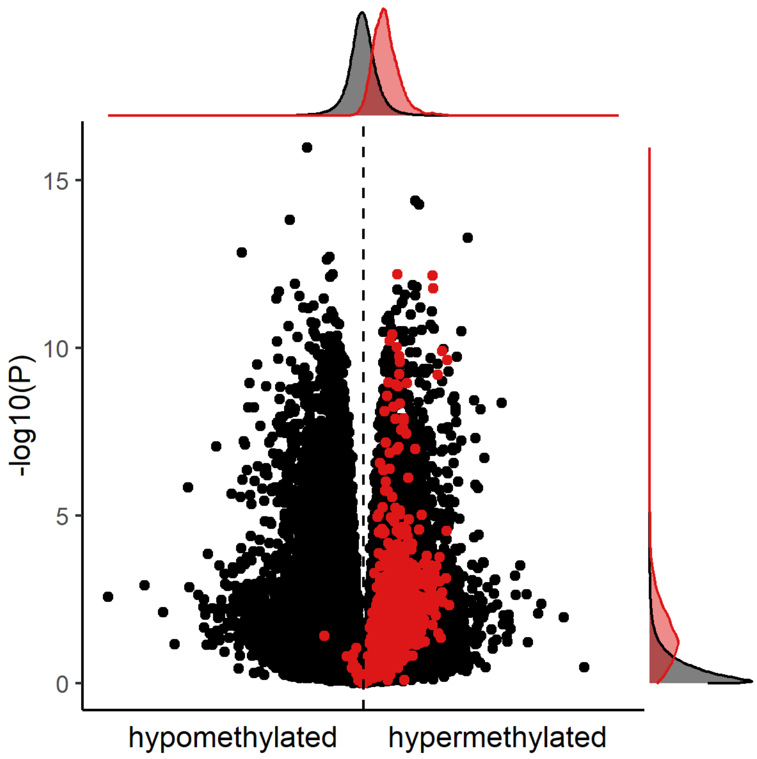

Supplementary Figure 6: Volcano plot showing association between CpG sites and *TET2* VAF in 44 individuals with *TET2* mutated CHIP. Red highlighted dots are the 2,741 sites that were significantly hypermethylated in CHIP (Figure 1C). P values and effect size estimates derived using a linear mixed effects regression with twin pair as random intercept. P values are two-sided and not adjusted for multiple comparisons.

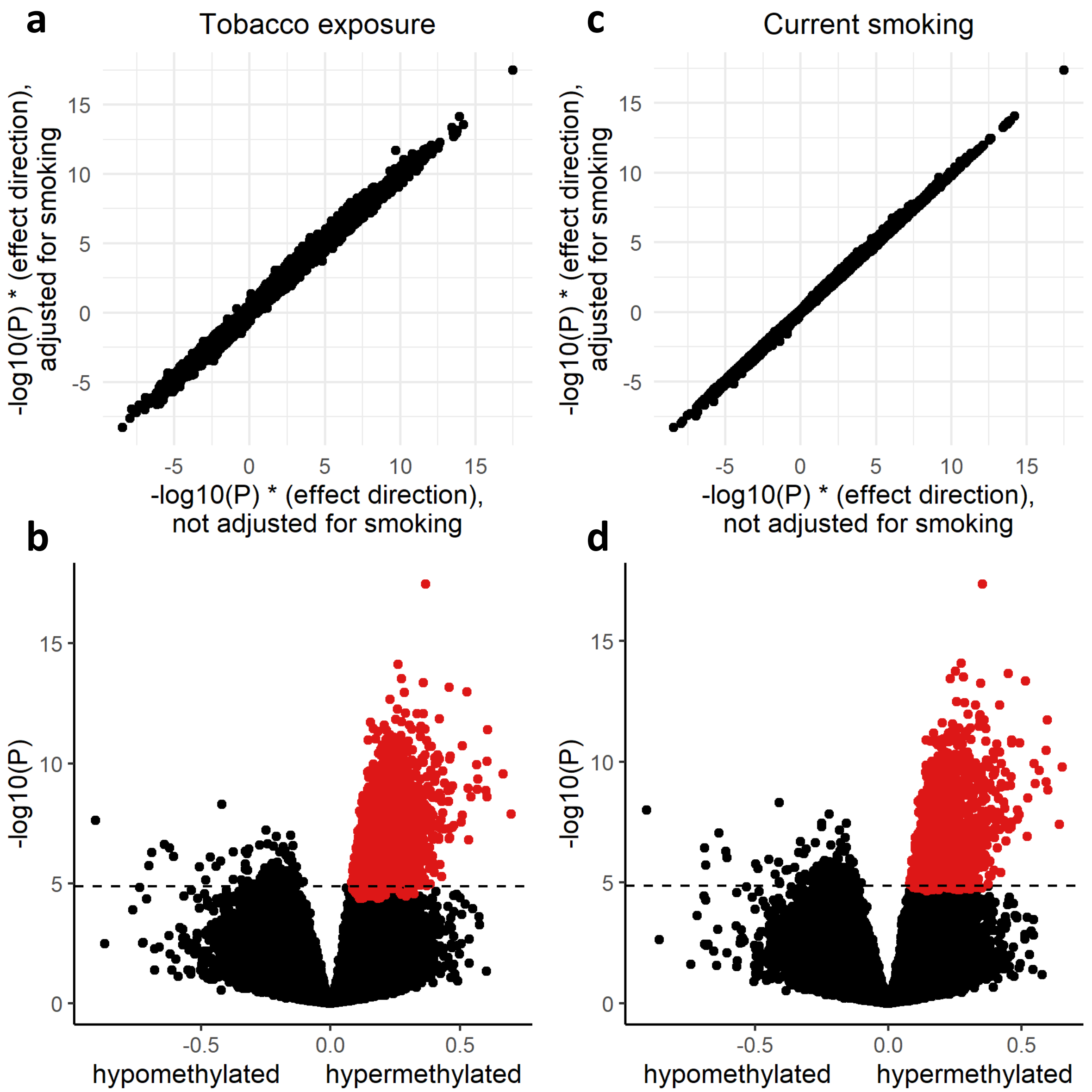

Supplementary Figure 7: Sensitivity analyses of *TET2* mutations with adjustment for smoking. **a** P value distributions before and after adjustment for smoking, defined as cumulative tobacco exposure (in pack-years, divided into tertiles and with never-smokers as reference group). P values multiplied by effect direction (1 for positive/hypermethylated sites and -1 for negative/hypomethylated sites).**b**: Volcano plot of analyses adjusted for cumulative tobacco exposure, highlighted red dots are the 2,741 sites that were significantly hypermethylated in main analyses (not adjusted for smoking). Horizontal dashed line indicated P value cutoff of 1.4×10^{-5} **c**: Same as A but adjusted for current smoking status (yes/no) instead. **d**: Same as B but adjusted for current smoking status (yes/no) instead. P values and effect size estimates in all panels derived using a linear mixed effects regression with twin pair as random intercept. All P values are two-sided and not adjusted for multiple comparisons.

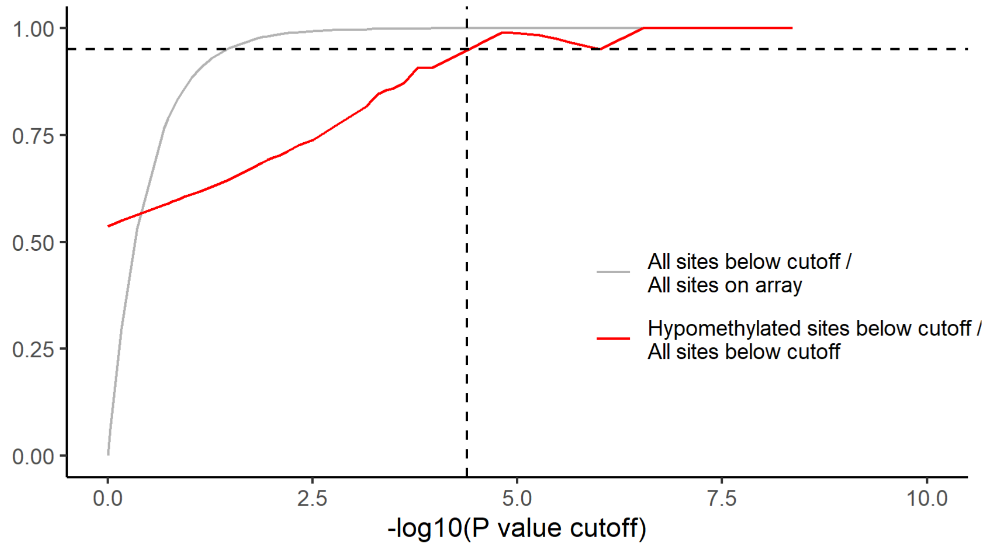

Supplementary Figure 8: Selection of P value cutoff in *DNMT3A*-specific analyses. Horizontal dashed line indicates 0.95. Vertical dashed line indicates $P = 5 \times 10^{-5}$, i.e. the cutoff below which 95% of sites are hypomethylated. P values and effect size estimates derived using a linear mixed effects regression with twin pair as random intercept. P values are two-sided and not adjusted for multiple comparisons.

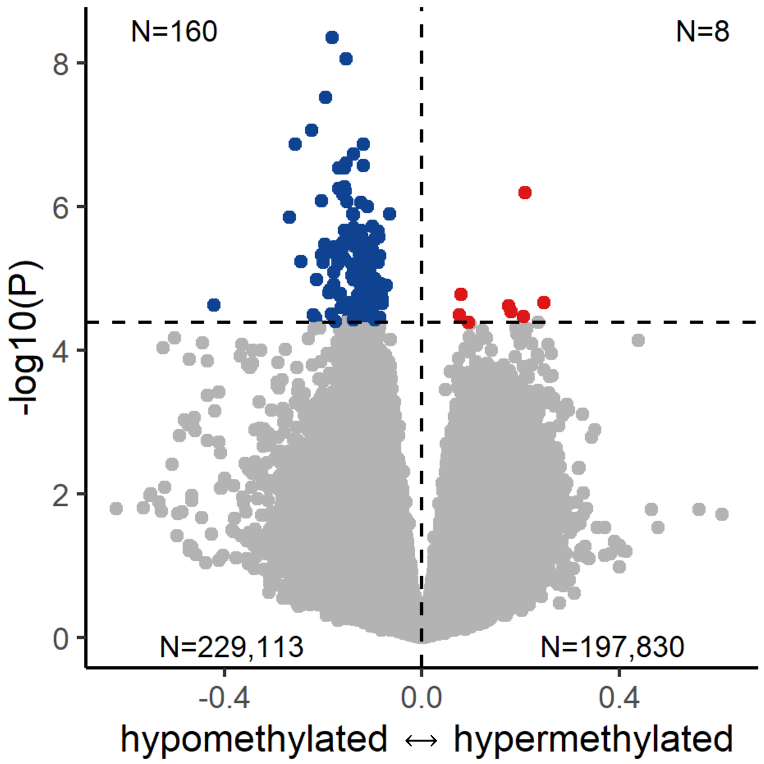

Supplementary figure 9: Volcano plot showing methylation differences between 55 *DNMT3A* mutant carriers and 189 non-CHIP carriers. Horizontal dashed line indicates $P = 4.1 \times 10^{-5}$. Numbers in corners indicate the number of CpG sites in each quadrant separated by dashed line. P values and effect size estimates derived using a linear mixed effects regression with twin pair as random intercept. P values are two-sided and not adjusted for multiple comparisons.

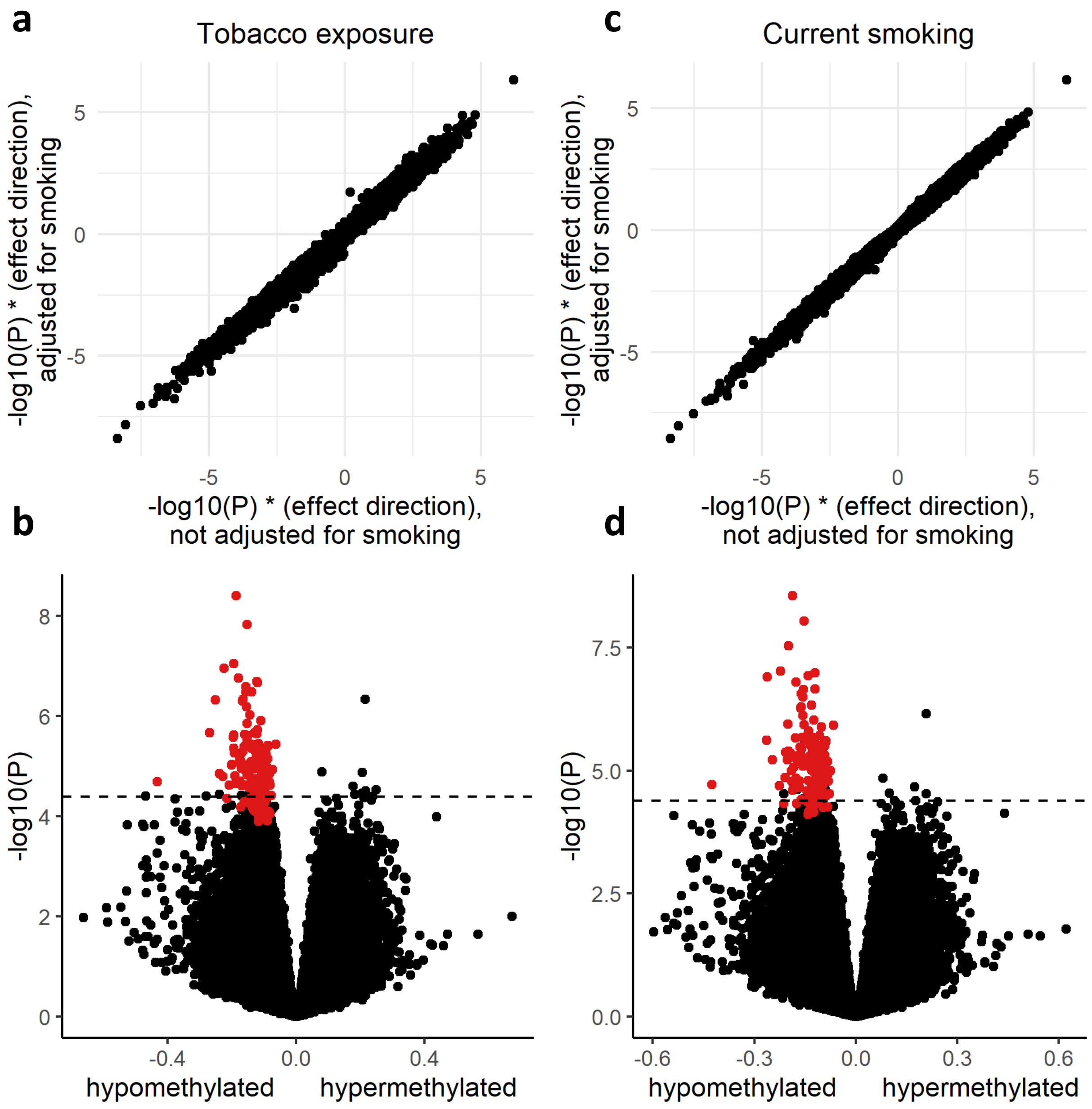

Supplementary Figure 10: Same as Supplementary Figure 7, but for *DNMT3A* analyses. P values and effect size estimates in all panels derived using a linear mixed effects regression with twin pair as random intercept. All P values are two-sided and not adjusted for multiple comparisons.

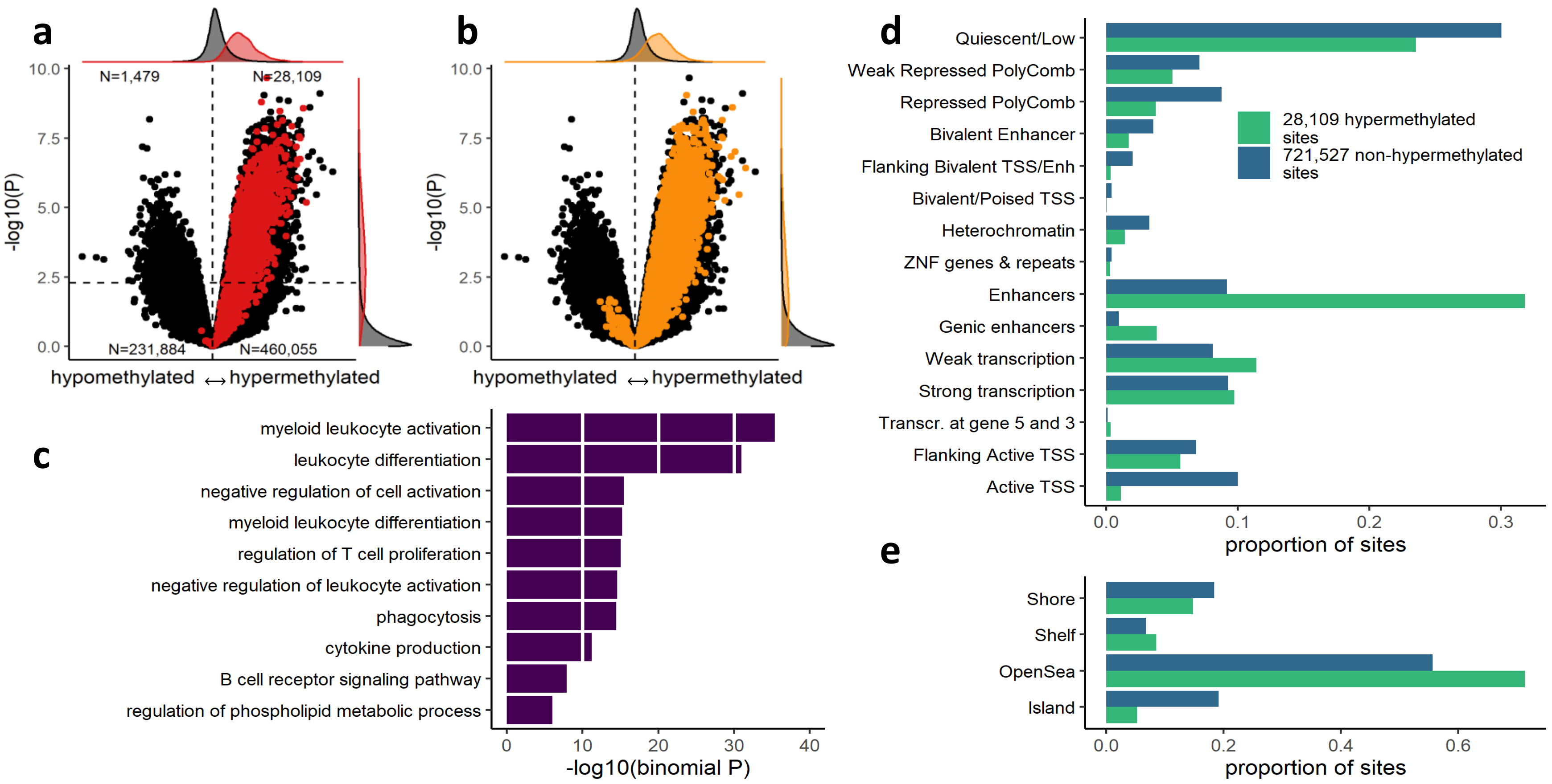

Supplementary Figure 11: Results of epigenome-wide analyses of TET2 mutations in MNC DNA from 10 CCUS patients with *TET2* mutations and 10 CCUS patients without *TET2* mutations. **a** Volcano plot of CCUS MNC results. Red dots indicate CpG sites from the set of 2,741 hypermethylated sites in CHIP (Fig 1C). Horizontal line shows P = 0.005, below which 95% of sites were hypermethylated. Margins display distributions of the 2,741 sites versus the rest of the EPIC array. Numbers in corners indicate the number of dots (both colors) in each quadrant separated by dashed lines. P values and effect size estimates derived using a linear model in Limma. P values are two-sided and not adjusted for multiple comparisons. **b** Volcano plot of CCUS MNC results (same as A), yellow dots indicate CpG sites from the set of 12,096 significantly hypermethylated sites in CCUS granulocyte DNA (upper right quadrant in Figure 3A). P values and effect size estimates derived using a linear

model in Limma. P values are two-sided and not adjusted for multiple comparisons. **c** Enrichment of Gene Ontology terms for the 5,000 most significantly hypermethylated sites in CCUS MNCs. P values derived using the region-based binomial test in GREAT and adjusted for multiple comparisons using the Bonferroni method. **d** Monocyte chromatin states for hypermethylated and non-hypermethylated sites. **E**: CpG island relations for hypermethylated and non-hypermethylated sites.

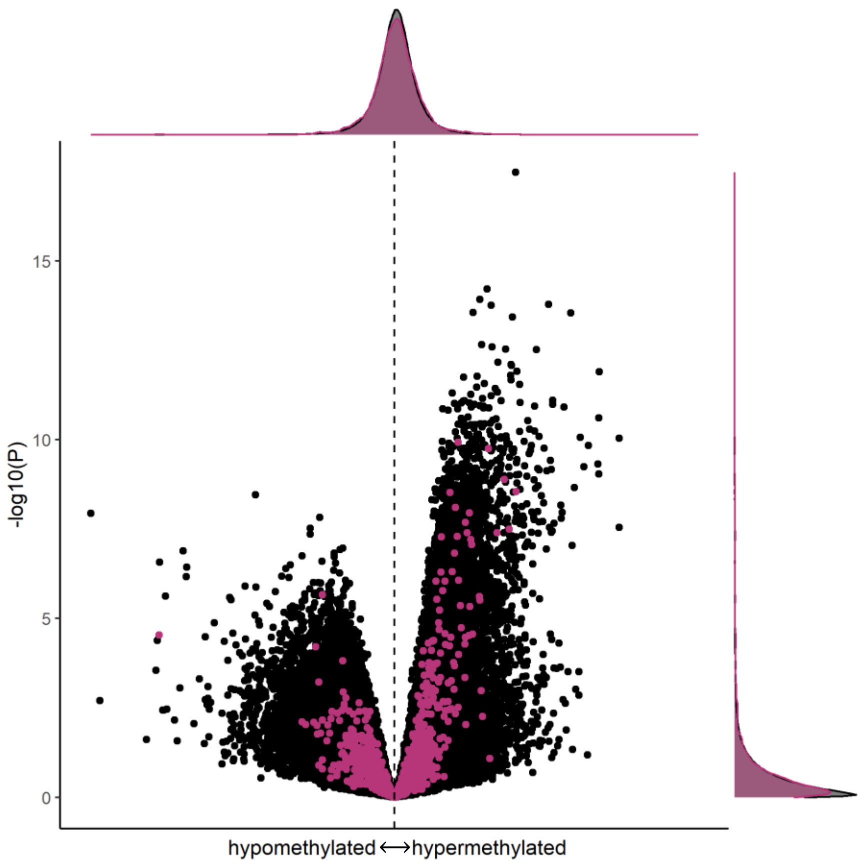

Supplementary figure 12: Volcano plot of results from epigenome-wide association studies on *TET2* mutations in CHIP. Purple dots indicate CpG sites that are also in the set of 2,000 hypermethylated sites in AML (Fig 5B). P values and effect size estimates derived using a linear mixed effects regression with twin pair as random intercept. P values are two-sided and not adjusted for multiple comparisons.

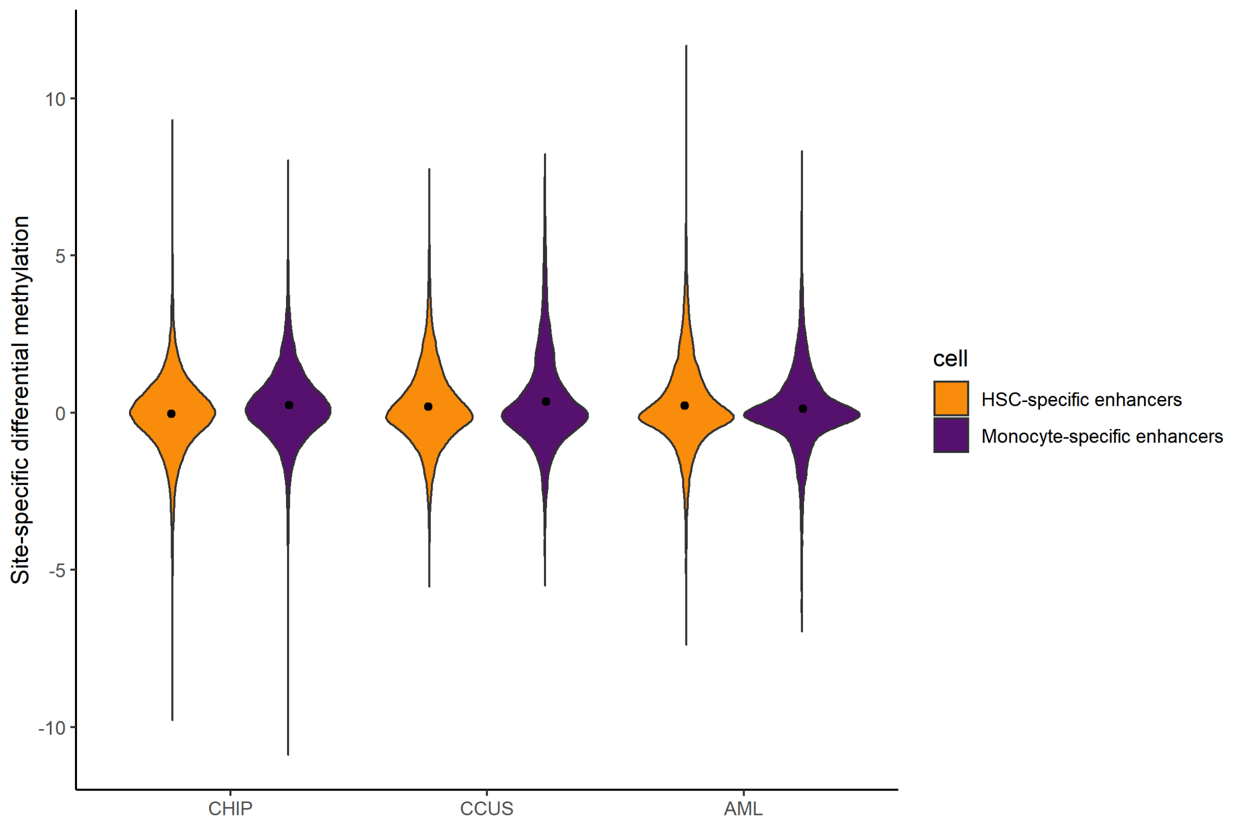

Supplementary Figure 13: Violin plots showing distrubution of scaled effect estimates mutations at all CpG sites located in HSC-specific (N = 47,748) and monocyte-specific enhancers (N = 37,518). Black dots represent means. See also Figure 5E for differences in means.

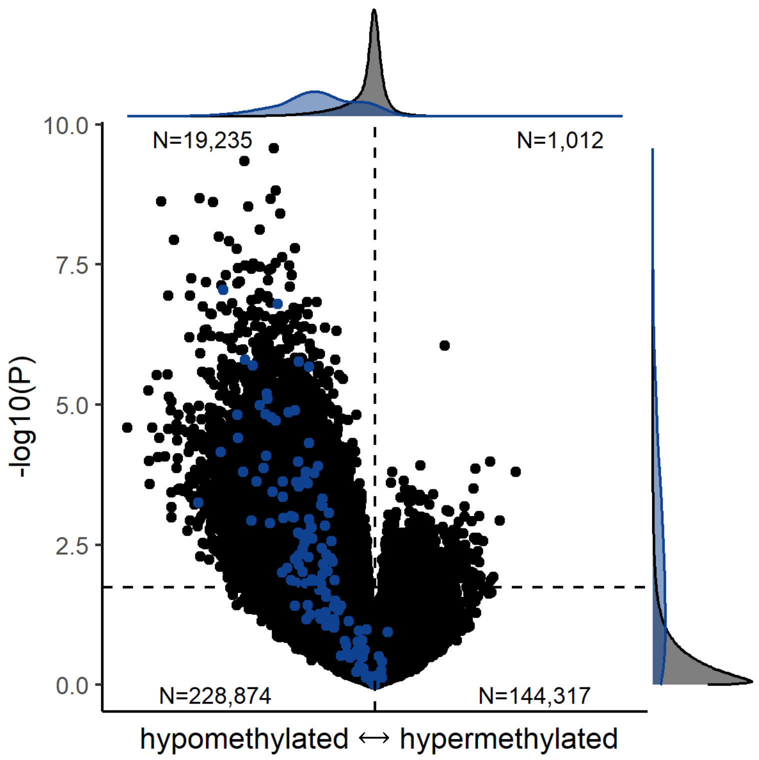

Supplementary Figure 14: Volcano plot of CpG site associations with 16 *DNMT3A* mutations in AML. Blue highlighted dots indicate sites that are in the set of 160 hypomethylated sites in *DNMT3A* mutated CHIP. P values and effect size estimates derived using a linear mixed effects regression with twin pair as random intercept. P values are two-sided and not adjusted for multiple comparisons.

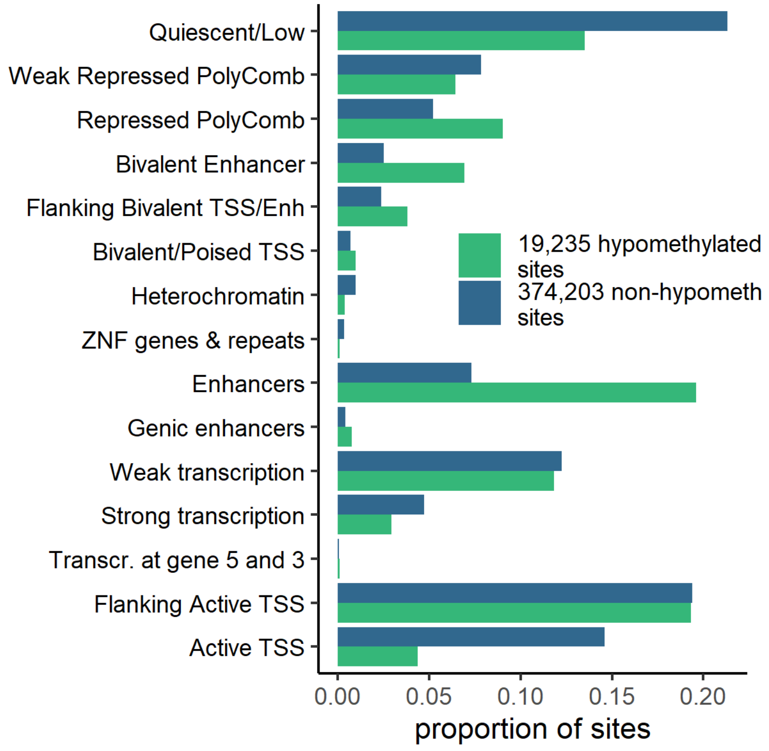

Supplementary Figure 15: HSC chromatin states for hypomethylated sites in *DNMT3A* mutated AML.

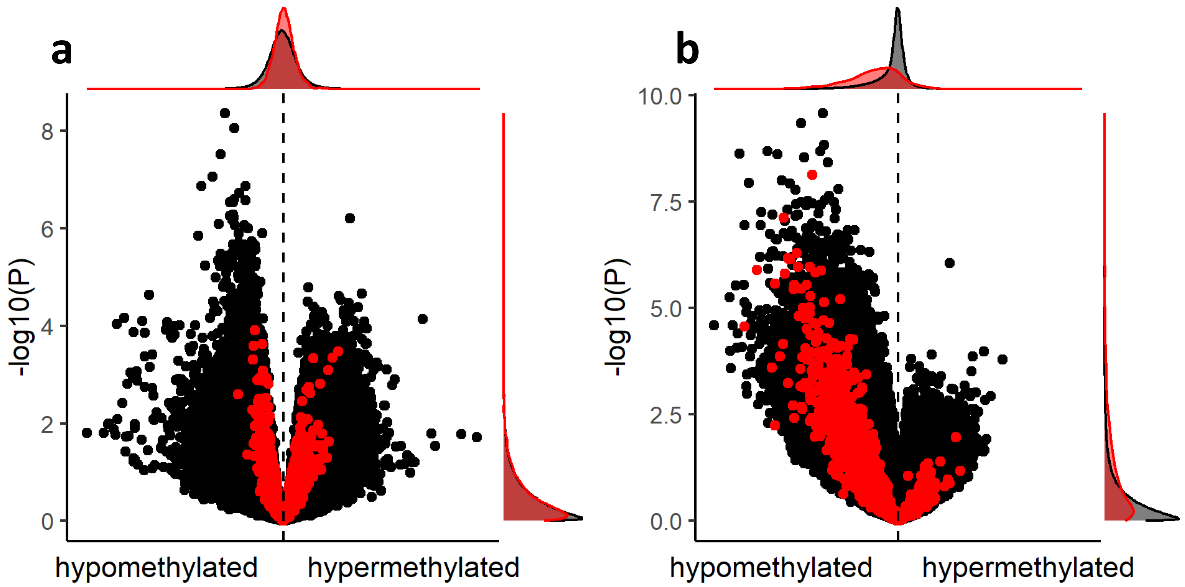

Supplementary Figure 16: Overlap between *DNMT3A* mutation-associated hypomethylation and *TET2* mutation-associated hypermethylation. **a** Volcano plot of CpG methylation associations with *DNMT3A* mutations in CHIP (similar to Supplementary Figure 10). Highlighted red dots indicate the 2,741 hypermethylated sites in *TET2* mutated CHIP. P values and effect size estimates derived using a linear mixed effects regression with twin pair as random intercept. P values are two-sided and not adjusted for multiple comparisons. **b** Volcano plot of CpG methylation associations with *DNMT3A* mutations in AML (similar to Supplementary Figure 13). Highlighted red dots indicate the 2,000 hypermethylated sites in *TET2* mutated AML. P values and effect size estimates derived using a linear model in Limma. P values are two-sided and not adjusted for multiple comparisons.

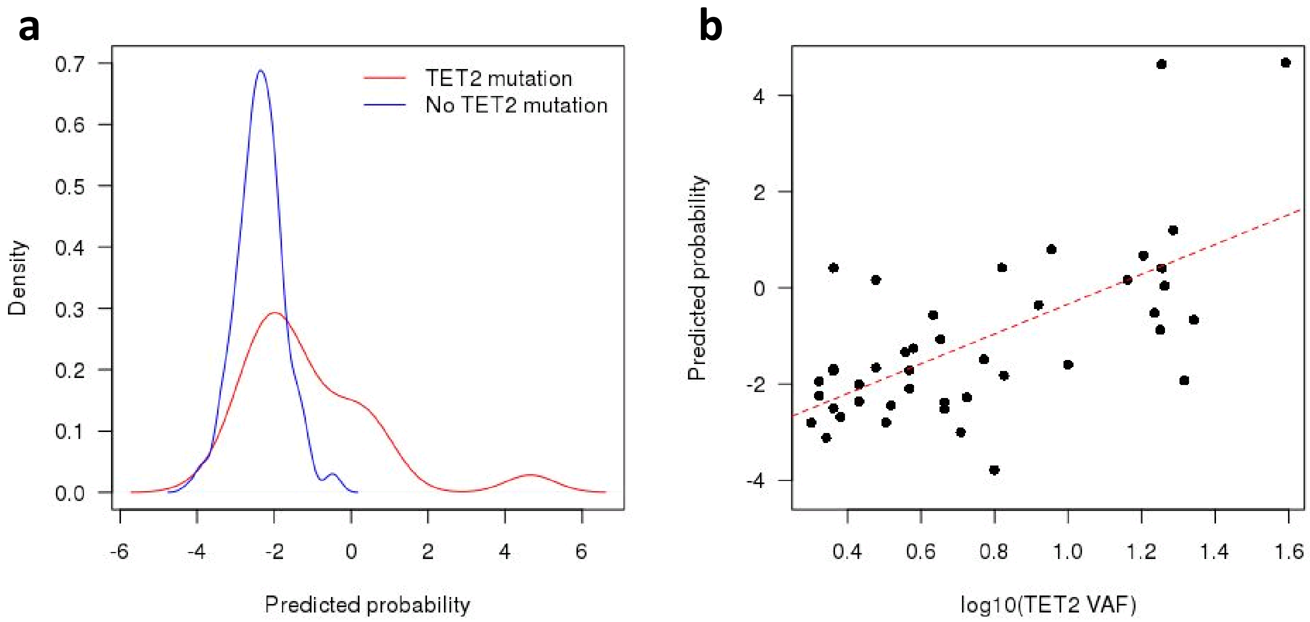

Supplementary figure 17 a Predicted probabilities of *TET2* mutations from leave-one-out cross validation of an elastic net logistic regression model in 44 *TET2* mutation carriers and 261 individuals without *TET2* mutations. **b** Predicted probabilities in individuals with *TET2* mutations by variant allele frequency, dashed red line indicates regression fit (P = 2.3×10^{-6}).

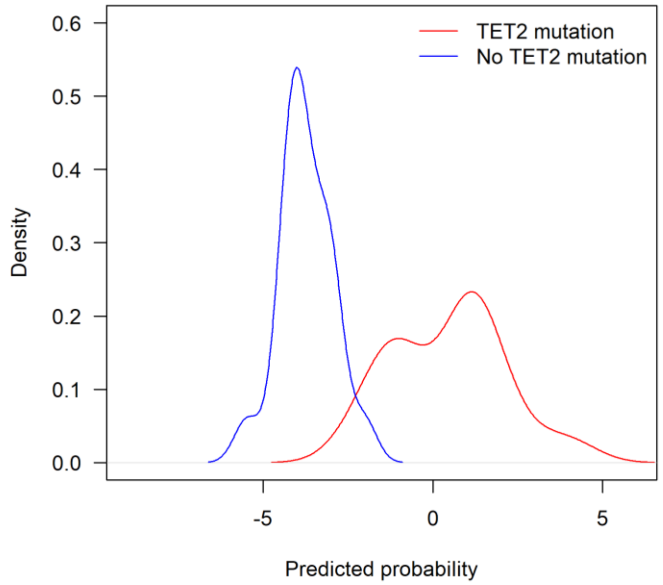

Supplementary figure 18: Predicted probability of *TET2* mutations in 33 CCUS patients and controls based on prediction model developed in CHIP cohort (Supplementary figure 8).

Supplementary References:

1. Dawoud, A. A. Z., Tapper, W. J. & Cross, N. C. P. Clonal myelopoiesis in the UK Biobank cohort: ASXL1 mutations are strongly associated with smoking. *Leukemia* **34**, 2660–2672 (2020).

2. Gao, X., Jia, M., Zhang, Y., Breitling, L. P. & Brenner, H. DNA methylation changes of whole blood cells in response to active smoking exposure in adults : a systematic review of DNA methylation studies. *Clin. Epigenetics* (2015) doi:10.1186/s13148-015-0148-3.

3. Pedersen, B. S., Schwartz, D. A., Yang, I. V & Kechris, K. J. Comb-p: software for combining, analyzing, grouping and correcting spatially correlated P-values. *Bioinformatics* **28**, 2986–2988 (2012).

4. Mallik, S. *et al.* An evaluation of supervised methods for identifying differentially methylated regions in Illumina methylation arrays. *Brief. Bioinform.* **20**, 2224–2235 (2019).

5. Houseman, E. A. *et al.* DNA methylation arrays as surrogate measures of cell mixture distribution. *BMC Bioinformatics* **13**, (2012).
