# Supplementary material for: TET2 mutations are associated with hypermethylation at key regulatory enhancers in normal and malignant hematopoiesis
Source: Nat Commun. 2021 Oct 18;12:6061. doi: 10.1038/s41467-021-26093-2 (PMC8523747; doi:10.1038/s41467-021-26093-2)
Supplement: Supplementary file 3 — Description of Additional Supplementary Files [file 41467_2021_26093_MOESM3_ESM.pdf]

Description of Additional Supplementary Files

File Name: Supplementary Data 1
Description: Regression results of differential methylation by *DNMT3A* mutation status for all CpG sites from the analyses of differential methylation in the 305 individuals in the CHIP cohort (55 with *DNMT3A* mutations).

File Name: Supplementary Data 2
Description: Regression results of differential methylation by *TET2* mutation status for all CpG sites from the analyses of differential methylation in the 305 individuals in the CHIP cohort (44 with *TET2* mutations).

File Name: Supplementary Data 3
Description: Regression results of differential methylation by *TET2* mutation status for all CpG sites from the analyses of differential methylation in the eight *TET2* mutated CCUS patients and five healthy controls (granulocyte data).

File Name: Supplementary Data 4
Description: Regression results of differential methylation by *TET2* mutation status for all CpG sites from the analyses of differential methylation in the 10 *TET2* mutated CCUS patients and 10 CCUS patients without *TET2* mutations (mononuclear cell data).

File Name: Supplementary Data 5

Description: Results of transcription factor motif enrichment analyses in both CHIP, CCUS, and AML. For each dataset is given enrichment (E)-value and rank by statistical significance. Only TF motifs that were statistically significant in at least one dataset is included.

File Name: Supplementary Data 6
Description: List of primers used for DNA sequencing library preparation.
