# Supplementary material for: TET2 mutations are associated with hypermethylation at key regulatory enhancers in normal and malignant hematopoiesis
Source: Nat Commun. 2021 Oct 18;12:6061. doi: 10.1038/s41467-021-26093-2 (PMC8523747; doi:10.1038/s41467-021-26093-2)
Supplement: Supplementary file 10 — Reporting Summary [file 41467_2021_26093_MOESM10_ESM.pdf]

nature portfolio

Corresponding author(s): Joachim Weischenfeldt
Kirsten Grønbæk

Last updated by author(s): Aug 15, 2021

Reporting Summary

Nature Portfolio wishes to improve the reproducibility of the work that we publish. This form provides structure for consistency and transparency in reporting. For further information on Nature Portfolio policies, see our Editorial Policies and the Editorial Policy Checklist.

Statistics

For all statistical analyses, confirm that the following items are present in the figure legend, table legend, main text, or Methods section.

n/a	Confirmed	
☐	☒	The exact sample size (n) for each experimental group/condition, given as a discrete number and unit of measurement
☐	☒	A statement on whether measurements were taken from distinct samples or whether the same sample was measured repeatedly
☐	☒	The statistical test(s) used AND whether they are one- or two-sided<br>*Only common tests should be described solely by name; describe more complex techniques in the Methods section.*
☐	☒	A description of all covariates tested
☐	☒	A description of any assumptions or corrections, such as tests of normality and adjustment for multiple comparisons
☐	☒	A full description of the statistical parameters including central tendency (e.g. means) or other basic estimates (e.g. regression coefficient) AND variation (e.g. standard deviation) or associated estimates of uncertainty (e.g. confidence intervals)
☐	☒	For null hypothesis testing, the test statistic (e.g. F, t, r) with confidence intervals, effect sizes, degrees of freedom and P value noted<br>*Give P values as exact values whenever suitable.*
☒	☐	For Bayesian analysis, information on the choice of priors and Markov chain Monte Carlo settings
☒	☐	For hierarchical and complex designs, identification of the appropriate level for tests and full reporting of outcomes
☒	☐	Estimates of effect sizes (e.g. Cohen's d, Pearson's r), indicating how they were calculated

Our web collection on statistics for biologists contains articles on many of the points above.

Software and code

Policy information about availability of computer code

Data collection	R versions 3.5.2 and 4.0.2
Data analysis	Bedtools v2.27.1<br>BWA-MEM v. 0.7.10<br>FreeBayes v.1.1.0<br>VarDict v.1.5.1<br>GREAT (http://bejerano.stanford.edu/great/public/html/index.php)<br>AME (https://meme-suite.org/meme/tools/ame)<br><br>R versions 3.5.2 and 4.0.2, including packages:<br>- Methylaid version 1.26.0<br>- Minfi version 1.34.0<br>- ChAMP version 2.18.3<br>- sva version 3.36.0<br>- lmerTest version 3.1-3<br>- Limma version 3.44.3<br>- glmnet version 4.1

For manuscripts utilizing custom algorithms or software that are central to the research but not yet described in published literature, software must be made available to editors and reviewers. We strongly encourage code deposition in a community repository (e.g. GitHub). See the Nature Portfolio guidelines for submitting code & software for further information.

Data

Policy information about availability of data

All manuscripts must include a data availability statement. This statement should provide the following information, where applicable:

- Accession codes, unique identifiers, or web links for publicly available datasets
- A description of any restrictions on data availability
- For clinical datasets or third party data, please ensure that the statement adheres to our policy

The raw and processed DNA sequencing and methylation data in the CHIP cohort generated in this study as well as other individual-level data from the Danish Twin Registry (DTR), including those derived from the Danish National Patient Registry, are are only available under restricted access, since these data are considered sensitive personal data according to Danish Law and the European Union General Data Protection Regulation (GDPR) and thus cannot be shared with third-parties without prior approval. To access the CHIP dataset, an application must be sent to tvilling@health.sdu.dk. The application will then be processed by the Scientific Board at DTR, and subsequently by Legal Services at the University of Southern Denmark (SDU-RIO) or the Danish Data Protection Agency (DDPA). Access can only be granted for research purposes, and only if a data processor or data transfer agreement can be made in accordance with Danish and European law at the given time. The expected timeframe from request to decision is approximately six weeks at the DTR and a few months at the SDU-RIO and DDPA.

The raw and processed DNA sequencing and methylation data on CCUS patients generated in this study are available under restricted access for the same reasons as stated for the CHIP dataset. To access the CCUS dataset, an application must be sent to Kirsten.Groenbaek@regionh.dk. Access can only be granted for research purposes, and only if a data processor or data transfer agreement can be made in accordance with Danish and European law at the given time. The expected timeframe from response until access is granted is approximately six months.

The chromatin state dataset for monocytes and HCSs used in this study are available in the Roadmap Epigenomics database under epigenome ids E029 and E035 (core 15-state model) [https://egg2.wustl.edu/roadmap/web_portal/chr_state_learning.html].

The transcription factor binding models dataset used in this study is available in the HOCOMOCO database (full dataset) [https://hocomoco11.autosome.ru/human/mono?full=false].

The TCGA LAML DNA sequencing and methylation datasets used in this study are available from the Genomic Data Commons Data Portal under project ID TCGA-LAML [https://portal.gdc.cancer.gov/].

The ExAC dataset (version 0.3) used in this study is available from the UCSC Genome Browser (https://hgdownload.soe.ucsc.edu/gbdb/hg19/ExAC/ExAC.r0.3.sites.vep.hg19.vcf.gz).

The 1000 Genomes dataset (Phase 3 v5c) used in this study is available from the 1000 Genomes web page (http://ftp.1000genomes.ebi.ac.uk/vol1/ftp/release/20130502/ALL.wgs.phase3_shapeit2_mvncall_integrated_v5c.20130502.sites.vcf.gz).

The TOPMED dataset (Freeze 3) used in this study is available from the BRAVO variant browser (https://bravo.sph.umich.edu/freeze3a/hg19/download).

Source Data are provided with this paper. All results from analyses of differential methylation by DNMT3A or TET2 mutations in CHIP and CCUS (regression coefficients, P values, mean values in cases and controls) including lists of the 2,741 and 12,096 top CpG sites in CHIP and CCUS and the complete results from the TF motif enrichment analyses are provided as Supplementary Data with this paper.

Field-specific reporting

Please select the one below that is the best fit for your research. If you are not sure, read the appropriate sections before making your selection.

☒ Life sciences ☐ Behavioural & social sciences ☐ Ecological, evolutionary & environmental sciences

For a reference copy of the document with all sections, see nature.com/documents/nr-reporting-summary-flat.pdf

Life sciences study design

All studies must disclose on these points even when the disclosure is negative.

Sample size	No sample-size calculations were performed. We used all the samples available to us.
Data exclusions	Four samples from the twin dataset were excluded due to outlier status in principal components analysis. These samples were extreme outliers in the first seven principal components, most likely indicating measurement error/artifacts. If not excluded, these samples would likely have caused biased results and violations of the assumptions behind the statistical models. The use of PCA as a QC measure and exclusion criterion was decided before the study was carried out.
Replication	The findings in the twin dataset were reproduced and extended in two separate CCUS datasets.
Randomization	This study did not test an intervention/experiment. It would not be possible to randomize humans to TET2 mutations.
Blinding	We did not perform any group allocation, and thus blinding was not possible.

Reporting for specific materials, systems and methods

We require information from authors about some types of materials, experimental systems and methods used in many studies. Here, indicate whether each material, system or method listed is relevant to your study. If you are not sure if a list item applies to your research, read the appropriate section before selecting a response.

Materials & experimental systems

n/a	Involved in the study
☒	☐ Antibodies
☒	☐ Eukaryotic cell lines
☒	☐ Palaeontology and archaeology
☒	☐ Animals and other organisms
☐	☒ Human research participants
☒	☐ Clinical data
☒	☐ Dual use research of concern

Methods

n/a	Involved in the study
☒	☐ ChIP-seq
☒	☐ Flow cytometry
☒	☐ MRI-based neuroimaging

Human research participants

Policy information about studies involving human research participants

Population characteristics	305 elderly monozygotic and dizygot twins from the population-based Danish Twin Registry. Age 73-94, 96 men and 209 women.<br>25 CCUS patients treated at Danish hematological departments. Age 60-82, 11 men and 114 women.<br>8 healthy controls (CCUS granulocyte dataset), elderly people who underwent hip arthroplasty surgery. Age 55-65, 4 men and 4 women.
Recruitment	Twins were recruited by invitation (all twins aged 75 years and older, alive and residing in Denmark in January 1995 were invited to participate in the study). Twins were included in consecutive waves with very high acceptance rates of 70-80% (PMID 31544734). Studies of responders vs. non-responders in the LSADT cohort have generally found very few relevant differences (PMID 10848141). As with all population-based studies, a selection for healthier individuals cannot be excluded, but we find no reason to assume that this selection would affect individuals differently depending on TET2 mutation status.<br>CCUS patients were recruited by the treating physician or a project nurse at the treating department.<br>Healthy controls were recruited at the treating department.<br>The fact that the healthy controls were all patients undergoing hip surgery could theoretically introduce a bias in the analyses on granulocyte data. However, since the results were replicated in the CCUS MNC data, in which all samples were from CCUS patients, it is unlikely that this selection has introduced any relevant bias.
Ethics oversight	The twin study was approved by The Regional Committees on Health Research Ethics for Southern Denmark<br>The study that recruited the CCUS patients and healthy controls was approved by the Danish National Committee on Research Ethics.

Note that full information on the approval of the study protocol must also be provided in the manuscript.
